# *Parachlamydia acanthamoebae* Infection and Abortion in Small Ruminants

**DOI:** 10.3201/eid1412.080582

**Published:** 2008-12

**Authors:** Silke Ruhl, Genevieve Goy, Nicola Casson, Rudolf Thoma, Andreas Pospischil, Gilbert Greub, Nicole Borel

**Affiliations:** University of Zurich, Zurich, Switzerland (S. Ruhl, A. Pospischil, N. Borel); University of Lausanne, Lausanne, Switzerland (G. Goy, N. Casson, G. Greub); Cantonal Laboratory of Veterinary Bacteriology, Chur, Switzerland (R. Thoma)

**Keywords:** Abortion, Chlamydiales, Parachlamydia, small ruminants, letter

**To the Editor:** Abortion in ruminants is of worldwide economic importance. Moreover, several abortigenic agents have a zoonotic potential, i.e., *Brucella abortus, Coxiella burnetii,* and *Chlamydophila abortus.* *C. abortus*, which causes ovine enzootic abortion, may also infect pregnant women who have had contact with *C. abortus*–infected sheep and goats, and such infection can lead to miscarriage (*1*).

*Parachlamydia acanthamoebae* (*2*) is a *Chlamydia*-related organism considered as an emerging agent of pneumonia in humans. Recently, we reported its role in the setting of bovine abortion (*3*). Here, we investigated the prevalence of *C. abortus* and *P. acanthamoebae* infections in abortions in small ruminants.

Formalin-fixed placenta, fetal lung and liver, or both, were available from abortion products from 144 goats and 86 sheep (n = 211). These specimens had previously been investigated for several abortigenic agents (*4*). Placentas and fetal organs were analyzed by histopathologic examination and by specific real-time PCR and immunohistochemical protocols that detect members of the *Chlamydiaceae* family and *P. acanthamoebae.*

DNA from paraffin blocks was extracted as described (*5*) by using the DNeasy Tissue kit (QIAGEN, Hilden, Germany). The real-time PCR for *Chlamydiaceae* was conducted on an ABI 7500 (Applied Biosystems, Foster City, CA, USA) by using a modified version of Everett’s PCR (*6*). Primers Ch23S-F (5′-CTGAAACCAGTAGCTTATAAGCGGT-3′), Ch23S-R (5′-ACCTCGCCGTTTAACTTAACTCC-3′), and probe Ch23S-p (5′-FAM-CTCATCA TGCAAAAGGCACGCCG-TAMRA-3′) were used to amplify and detect a 111-bp product specific for members of the family *Chlamydiaceae*. Chlamydial species identification of real-time PCR positive cases was performed with the ArrayTube Microarray (Clondiag, Jena, Germany) as described (*7*).

The *Parachlamydia-*specific real-time PCR was performed with the ABI Prism 7000 sequence detection system (Applied Biosystems), as reported (*8*). This PCR is genus-specific, as demonstrated by the absence of PCR positivity with DNA extracted from other *Parachlamydiaceae* (*Protochlamydia* spp*.*/*Neochlamydia hartmannellae).* To confirm positive results, another specific PCR, which targeted the *tlc* gene, was performed (*9*).

Paraffin sections from specimens positive in real-time PCR were further examined by immunohistochemical tests. A *Chlamydiaceae*-specific mouse monoclonal antibody directed against the chlamydial lipopolysaccharide (Progen, Heidelberg, Germany) and a specific mouse polyclonal antibody against *Parachlamydia* spp. was used as described (*3**,**5**,**10*). These antibodies were applied at dilutions of 1:200 and 1:1,000, respectively. Detection was performed with a detection kit (ChemMate; Dako, Glostrup, Denmark). Antigen retrieval was performed by enzyme digestion for 10 minutes (Pronase; Dako) for the *Chlamydiaceae* antibody and repeated microwave treatment in citrate buffer (ChemMate; Dako) for the *Parachlamydia* antibody, respectively. Double immunohistochemical labeling was performed on the sheep abortion specimen identified as simultaneously infected with *Chlamydiaceae* and *Parachlamydia* spp. Immunohistochemical analysis for both pathogens was performed subsequently by using diaminobenzidine as substrate for the *Chlamydiaceae* antibody (brown labeling) and by using 3-amino-9-ethylcarbazole as substrate for the *Parachlamydia* antibody (red labeling). Specificity of PCR and immunohistochemical tests for *Chlamydiaceae* and *Parachlamydia* spp., respectively, was assessed by using negative control placentas taken from 2 healthy ruminants (both specimens were negative in all tests).

Results of real-time PCR showed that 55 (26.1%) of 211 specimens were positive for *Chlamydiaceae*. All 55 cases could be identified as *C. abortus* by ArrayTube Microarray (Clondiag). Of these, 42 (76.4%) could be confirmed by immunohistochemical analysis with the anti-*Chlamydiaceae* antibody.

Of the 211 specimens, only 2 (0.9%) were positive for *Parachlamydia* spp. by real-time PCR, and both cases could be confirmed by immunohistochemical testing with the parachlamydial antibody. These 2 specimens were negative for other common abortigenic agents such as *Toxoplasma gondii, C. burnetii,* and border disease virus (data not shown). One case was recorded among the 144 goat samples investigated. This placenta displayed necrotizing placentitis and was positive for *Parachlamydia* spp. by 16S rRNA-specific real-time PCR (cycle threshold [Ct] 40.5) and immunohistochemical testing, but negative for *Chlamydiaceae*. Results of this PCR was confirmed by another PCR, targeting the *tlc* gene (Ct 36.7), which excluded false-positive results because of amplicon contamination.

The second case was identified among the 86 sheep investigated. Placenta and fetal lung and liver exhibited necrotizing placentitis and vasculitis (Figure, panel **A**), interstitial pneumonia (Figure, panel **B**), and mixed cellular periportal hepatitis. Fetal liver was negative by parachlamydial 16S rRNA real-time PCR and immunohistochemical analysis, but the fetal lung was positive by parachlamydial 16S rRNA real-time PCR (Ct 40.7) and immunohistochemical tests (Figure, panel **C**), but negative with the *tlc* PCR. Fetal lung and liver were positive by real-time PCR for *Chlamydiaceae* (mean Ct for both organs 36.7), but negative by immunohistochemical tests. The placenta was positive for *Chlamydiaceae* by immunohistochemical tests and real-time PCR (mean Ct 23.3), and *C. abortus* was identified by ArrayTube Microarray. Brown (*Chlamydiaceae*) and red (*Parachlamydia* spp.) granular reaction was demonstrated within the necrotic lesions of the placenta by double immunohistochemical labeling (Figure, panel **D**).

**Figure:**
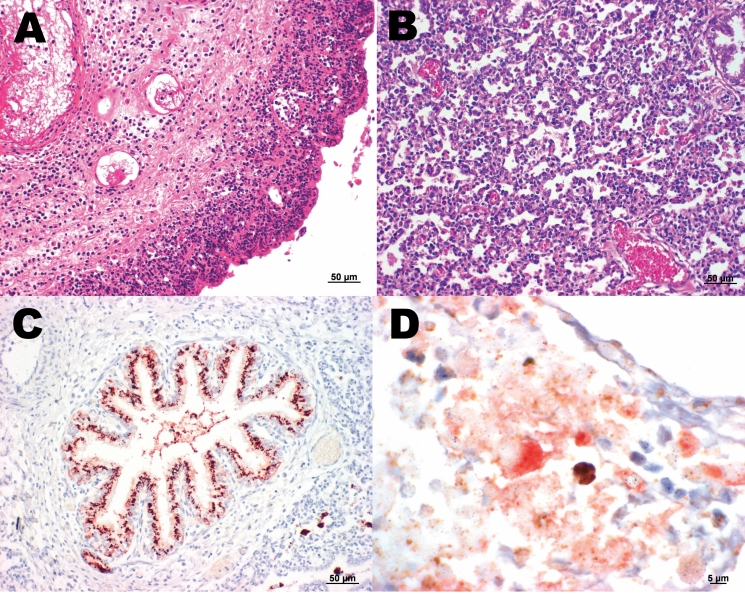
A) Sheep placenta positive by real-time PCR and immunohistochemistry for *Parachlamydia* spp. and *Chlamydiaceae. Chlamydophila abortus* was identified by ArrayTube Microarray. Necrotizing placentitis and vasculitis are shown (hematoxylin and eosin stain; magnification ×200). B) Fetal lung of the sheep abortion specimen positive by real-time PCR and immunohistochemical tests for *Parachlamydia* spp. and *Chlamydiaceae*; interstitial pneumonia is shown (hematoxylin and eosin stain; magnification ×200). C) Fetal lung that was positive by real-time PCR and immunohistochemical testing for *Parachlamydia* spp. Positive granular material can be seen within the lung tissue. Antigen detection (immunohistochemistry) was carried out with a polyclonal antibody directed against *Parachlamydia* spp. 3-amino-9-ethylcarbazole/peroxidase method (hematoxylin counterstain; magnification ×200). D) Double immunohistochemical labeling of the sheep placenta that was positive by real-time PCR and immunohistochemical tests for *Chlamydiaceae* and *Parachlamydia* spp. The simultaneous presence of *Chlamydiaceae* and *Parachlamydia* spp. granular reaction is shown within necrotic trophoblastic epithelium and neutrophilic exudate (diaminobenzidine/AEC/peroxidase method, hematoxylin counterstain; magnification ×1,000).

We report *Parachlamydia* infection in small ruminant abortion. *C. abortus* and *Parachlamydia* spp. were simultaneously present in an aborted sheep placenta. *Parachlamydia* spp. could be further detected in the lung of the aborted sheep fetus by real-time PCR and immunohistochemistry. *Parachlamydia* was also detected in a goat placenta. Thus, *Parachlamydia* spp. should be considered as a new abortigenic agent in sheep and goats. Persons in contact with small ruminants should be informed about the zoonotic potential of these abortigenic agents.
